# Impact of Age and Comorbidity on Choice and Outcome of Two Different Treatment Options for Patients with Potentially Curable Esophageal Cancer

**DOI:** 10.1245/s10434-019-07181-6

**Published:** 2019-02-04

**Authors:** Z. Faiz, M. van Putten, R. H. A. Verhoeven, J. W. van Sandick, G. A. P. Nieuwenhuijzen, M. J. C. van der Sangen, V. E. P. P. Lemmens, B. P. L. Wijnhoven, J. T. M. Plukker

**Affiliations:** 1 Department of Surgery, University of Groningen, University Medical Center Groningen, Groningen, The Netherlands; 2 Department of Research, Netherlands Comprehensive Cancer Organization, Utrecht, The Netherlands; 3 Department of Surgery, Netherlands Cancer Institute, Antoni van Leeuwenhoek Hospital, Amsterdam, The Netherlands; 4 Department of Surgery, Catharina Hospital Eindhoven, Eindhoven, The Netherlands; 5 Department of Radiotherapy, Catharina Hospital Eindhoven, Eindhoven, The Netherlands; 6 Department of Public Health, Erasmus MC University Medical Center Rotterdam, Rotterdam, The Netherlands; 7 Department of Surgery, Erasmus MC University Medical Center Rotterdam, Rotterdam, The Netherlands

## Abstract

**Purpose:**

This study was designed to assess the impact of age and comorbidity on choice and outcome of definitive chemoradiotherapy (dCRT) or neoadjuvant chemoradiotherapy plus surgery.

**Methods:**

In this population-based study, all patients with potentially curable EC (cT1N+/cT2-3, TX, any cN, cM0) diagnosed in the South East of the Netherlands between 2004 and 2014 were included. Kaplan–Meier method with log-rank tests and multivariable Cox regression analysis were used to compare overall survival (OS).

**Results:**

A total of 702 patients was included. Age ≥ 75 years and multiple comorbidities were associated with a higher probability for dCRT (odds ratio [OR] 8.58; 95% confidence interval [CI] 4.72–15.58; and OR 3.09; 95% CI 1.93–4.93). The strongest associations were found for the combination of hypertension plus diabetes (OR 3.80; 95% CI 1.97–7.32) and the combination of cardiovascular with pulmonary comorbidity (OR 3.18; 95% CI 1.57–6.46). Patients with EC who underwent dCRT had a poorer prognosis than those who underwent nCRT plus surgery, irrespective of age, number, and type of comorbidities. In contrast, for patients with squamous cell carcinoma with ≥ 2 comorbidities or age ≥ 75 years, OS was comparable between both groups (hazard ratio [HR] 1.52; 95% CI 0.78–2.97; and HR 0.73; 95% CI 0.13–4.14).

**Conclusions:**

Histological tumor type should be acknowledged in treatment choices for patients with esophageal cancer. Neoadjuvant chemoradiotherapy plus surgery should basically be advised as treatment of choice for operable esophageal adenocarcinoma patients. For patients with esophageal squamous cell carcinoma with ≥ 2 comorbidities or age ≥ 75 years, dCRT may be the preferred strategy.

For potentially curable esophageal cancer (EC), radical surgery after neoadjuvant chemoradiotherapy (nCRT) has been the standard of care in the Netherlands since 2008.^1^ However, surgery is associated with postoperative morbidity in up to 60% of patients with a 90-day mortality rate of 7–13%.^2^–^6^ In general, comorbidity and older age are related to early postoperative mortality after gastrointestinal cancer surgery.^7^ A less aggressive treatment approach may be considered in these patients.^8^ Definitive chemoradiotherapy (dCRT) is an alternative curative intended treatment option in elderly patients and in patients with severe comorbidities.^3^,^9^–^11^ Similar survival rates have been reported after chemoradiotherapy with or without surgery for patients with esophageal squamous cell carcinoma (ESCC).^11^,^12^ In patients with esophageal adenocarcinoma (EAC), surgery is recommended unless there is a high risk for threatening postoperative complications and/or mortality.^13^–^16^

Long-term outcome data following dCRT for potentially curable EC are scarce and guidelines for selecting the appropriate treatment in patients with severe comorbidity and older age are not available.^13^,^17^ The purpose of this population based, retrospective study was to assess the impact of age and comorbidity on the choice of curative intended treatment and long-term overall survival among patients with potentially curable esophageal cancer.

## Patients and Methods

Data from all patients with a primary esophageal cancer (CI 5.1–5.9), diagnosed between 2004 and 2014 in the South East of the Netherlands, were obtained from the population-based nationwide Netherlands Cancer Registry (NCR). Data from this region was used, because data on comorbidities was not routinely registered by the NCR in other parts of the Netherlands during the study period. Trained data managers of the NCR routinely extract information on diagnosis, tumor stage, comorbidity, and treatment from the medical hospital records, using a strict registration and coding manual. Tumors were clinically staged according to the UICC/AJCC TNM classification that was valid at the time of diagnosis.

**Fig. 1:**
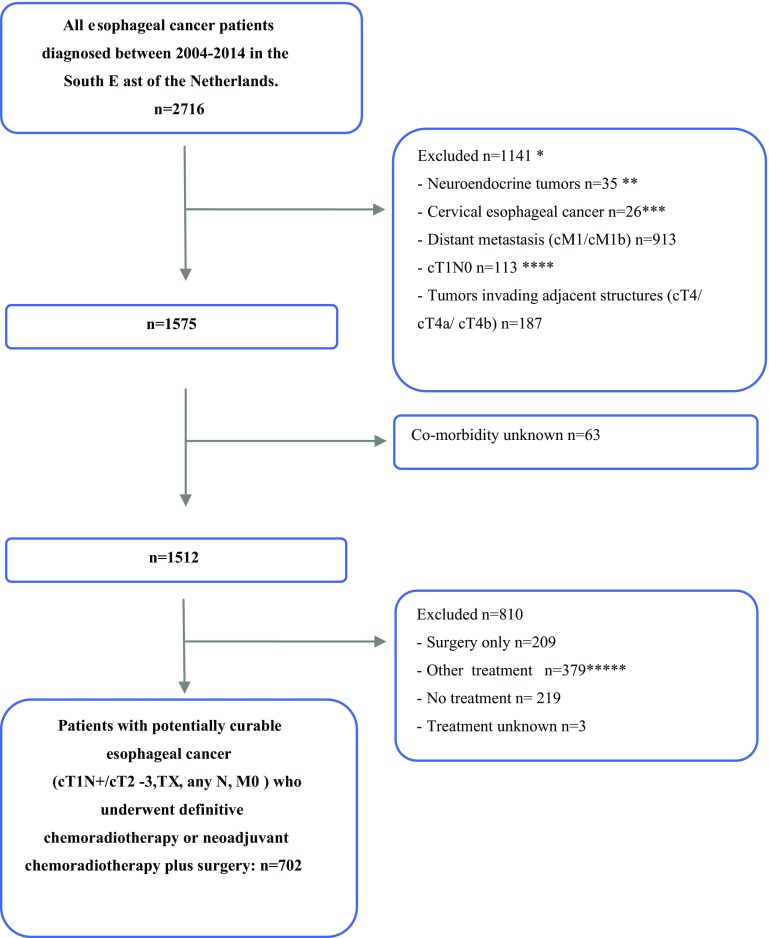
Flowchart of study population. *The sum of excluded patients per exclusion criteria is larger than the total number of excluded patients because some patients met two exclusion criteria. **Lymphoma, melanoma were already excluded. ***Not eligible for surgery. ****Eligible for endoscopic resection. *****74% underwent radiotherapy only

Patients with potentially curable EC (cT1N+/cT2-3, TX, any cN, cM0) and treated with dCRT or nCRT plus surgery were eligible for this study (Fig. 1). Patients were classified as cTX when the tumor could not be sufficiently subcategorized, for example due to an obstructing tumor that could not be passed during endoscopic ultrasonography. Patients were considered potentially curable if they had clinically no distant metastasis (cM0 according to TNM-7 and cM1a, i.e., positive coeliac nodes, according to TNM-6), and no tumor invasion into surrounding organs (no cT4 according to TNM-6 and no cT4a or cT4b according to TNM-7). Although patients with a cT4a tumor could theoretically be treated with curative intent, all cT4 tumors were excluded, because they were only distinguished after 2010 by TNM-7. For the analysis, patients with a cM1a tumor according to TNM-6 were categorized as having cN+ according to TNM-7. As of 2010, coding regulations to register a cM0 or cM1 status into the NCR were less strict than before 2010. As a consequence, since 2010, relatively more patients were registered with no (cM0) rather than unknown clinical distant metastases into the NCR. To account for this, we decided to include all patients with cMX. Patients with cervical esophageal cancer (CI 5.0) and those with a cT1N0 tumor were excluded, because surgery was not standard care in these patients. Patients who underwent palliative or other treatment were excluded from the analysis (Fig. 1).

In this study, neoadjuvant CRT with curative intent consisted of 5 cycles of carboplatin (area under the curve 2 mg/ml/min)/paclitaxel 50 mg/m^2^ and 41.4 Gy/1.8 Gy or occasionally 50.4 Gy/1.8 Gy radiotherapy followed by potentially curative surgery, based on the CROSS regimen.^1^,^18^ Definitive or primary CRT usually included concurrent chemotherapy (cisplatin/5-FU or carboplatin/paclitaxel) and radiotherapy > 50.4 Gy/1.8–2 Gy as first treatment in patients who were unable to undergo surgical resection.^19^,^20^ In the analysis, patients with primary intended nCRT of 41.4–50.4 Gy/1.8 Gy in whom additional surgical resection was denied because of deteriorated medical condition and potentially high risk for severe morbidity and mortality.

In the NCR, comorbidities were registered according to a slightly modified version of the Charlson comorbidity index.^21^ The Charlson comorbidity index is most widely used for recording comorbidity and was validated in various studies. Comorbidity was defined as life-shortening diseases that were present at the time of cancer diagnosis.^22^–^24^

The following groups of comorbidities were included in our analyses: pulmonary disease (COPD, emphysema, chronic bronchitis), cardiovascular disease (angina pectoris, myocardial infarction, cardiomyopathy, myocarditis, vascular disease, TIA, CVA), hypertension, diabetes mellitus, and previous invasive malignancies. Patients with no serious comorbidity in the medical file were registered as having no comorbidity. Patients were excluded if comorbidity status was not registered.

Statistics Netherlands developed an indicator of Socio-Economic Status Score (SES), using individual fiscal data based on the economic value of the home and household income. This SES indicator is provided at an aggregated level for each postal code (covering an average of 17 households). SES was categorized as low (deciles 1–3), medium (deciles 4–7), or high (deciles 8–10). A separate category was made for postal codes of care-providing institutions, because assigning SES for those living in nursing home or other care providing institutions is difficult.

### Statistics

Differences between patient groups were analysed by using Chi square tests. Multivariable logistic regression analyses were performed to examine the impact of clinicopathological factors on the choice of curative-intended treatment (dCRT vs. nCRT followed by surgery). Survival time was defined as time from 6 months after diagnosis until death or until February 2017 for patients who were still alive. Thus, patients who died within 6 months after diagnosis were excluded from survival analysis. This was done to deal with immortal time bias, i.e., the waiting period of 6–8 weeks between end of CRT and surgery in patients undergoing nCRT, because total treatment duration for those who underwent dCRT is shorter.^25^ Overall survival (OS) was calculated with the Kaplan–Meier analysis, and log-rank tests were performed to test for differences between groups. Multivariable survival analyses were performed using the Cox proportional hazards model (HR and 95% confidence intervals) to investigate the prognosis after dCRT versus nCRT plus surgery after adjustment for confounders. According to histological tumor type, separate models were performed for age categories, number of comorbidities, and for each type of comorbidity. All analyses were performed in SAS version 9.4, and two-sided *p* values < 0.05 were considered statistically significant.

## Results

### Clinicopathological Characteristics

A total of 702 patients was included in the study (Fig. 1). Neoadjuvant CRT with surgery was performed in 386 patients (55%) and dCRT in 316 patients (45%). Frequently reported comorbidities were cardiovascular disease (33%), hypertension (33%), pulmonary disease (15%), and diabetes (15%; Table 1). Most tumors were adenocarcinomas (65%) and in a locally advanced stage with cT3 (65%) and cN1-3 (60%). Approximately 81% of the patients were treated after 2008.

**Table 1:** Characteristics of esophageal cancer patients (cT1N+/cT2-3,TX, any cN, cM0) treated with definitive chemoradiotherapy or neoadjuvant CRT followed by surgery diagnosed in the South East of the Netherlands in the period 2004–2014 (*N* = 702)

	All patients (*N* = 702)	
	N	%
Treatment		
dCRT	316	45
nCRT + surgery	386	55
Gender		
Male	535	76
Female	167	24
Age (year)		
< 60	184	26
60–74	387	55
≥ 75	131	19
Number of comorbidities		
0	211	30
1	218	31
≥ 2	273	39
Type of comorbidity		
Cardiovascular	231	33
Pulmonary	108	15
Hypertension	232	33
Previous malignancies	72	10
Diabetes	102	15
Socioeconomic status		
Low	153	22
Intermediate	277	39
High	219	31
Care providing institution	21	3
Unknown	32	5
Tumor localization		
Proximal	38	5
Mid	92	13
Distal	544	77
Overlapping/not otherwise specified	28	4
Histology		
EAC	457	65
ESCC	230	33
Other/unknown	15	2
cT classification		
T1	6	< 1
T2	138	20
T3	455	65
TX	103	15
cN classification		
N0	259	37
N+	423	60
NX	20	3
Period of diagnosis		
2004–2008	133	19
2009–2014	569	81

*EAC* esophageal adenocarcinoma; *ESCC* esophageal squamous cell carcinoma; *dCRT* definitive chemoradiotherapy; *nCRT* neoadjuvant chemoradiotherapy

### Association Between Age and Treatment

Of the patients treated with nCRT and surgery, less than 8% (29/386 patients) were 75 years or older (Table 2), whereas 19% (60/316) of the patients treated with dCRT were younger than 60 years. Approximately 78% (102/131 patients) of the elderly (≥ 75 years) patients were treated with dCRT, whereas only 33% (60/184 patients) of the patients younger than 60 years underwent dCRT.

**Table 2:** Multivariable logistic regression analysis of clinicopathological factors upon the likelihood of treatment with dCRT versus nCRT followed by surgery among patients with esophageal cancer (cT1N+/cT2-3,Tx, any cN, cM0) diagnosed in the South East of the Netherlands in the period 2004–2014 (*N* = 702)

	Patients				*p* value	Multivariable analysis	
	dCRT (*n* = 316)		nCRT + surgery (*n* = 386)			dCRT versus nCRT + surgery	
	*N*	%	*N*	%		OR	95% CI
Gender					< 0.01		
Male	218	69	317	82		1.0	
Female	98	31	69	18		1.38	0.88–2.17
Age (year)					< 0.01		
< 60	60	19	124	32		1.0	
60–74	154	49	233	60		1.08	0.69–1.68
≥ 75	102	32	29	8		8.58	4.72–15.58
Number of comorbidities					< 0.01		
0	69	22	142	37		1.0	
1	87	28	131	34		1.34	0.84–2.15
≥ 2	160	51	113	29		3.09	1.93–4.93
Type of comorbidity^a^							
Cardiovascular	132	42	99	26	< 0.01	1.74	1.18–2.57
Pulmonary	63	20	45	12	< 0.01	2.08	1.28–3.38
Hypertension	118	37	114	30	0.03	1.40	0.95–2.06
Previous malignancies	44	14	28	7	< 0.01	1.55	0.86–2.80
Diabetes	60	19	42	11	< 0.01	2.39	1.45–3.92
Cardiovascular and pulmonary	34	11	17	4	< 0.01	3.18	1.57–6.46
Hypertension and diabetes	40	13	18	5	< 0.01	3.80	1.97–7.32
Socioeconomic status					0.05		
Low	79	25	74	19		1.0	
Intermediate	125	40	152	39		0.67	0.42–1.06
High	84	27	135	35		0.57	0.35–0.93
Care providing institution/unknown	28	9	25	6		0.72	0.34–1.55
Tumor localization					< 0.01		
Proximal/mid	98	31	32	8		1.0	
Distal	204	65	340	88		0.23	0.13–0.40
Overlapping/not otherwise specified	14	4	14	4		0.37	0.14–0.98
Histology^b^					< 0.01		
EAC	158	50	299	77		1.0	
ESCC	149	47	81	21		1.95	1.24–3.06
cT classification					< 0.01		
cT1-2	70	22	74	19		1.0	
cT3	184	58	271	70		0.66	0.42–1.03
cTX	62	20	41	11		1.34	0.72–2.48
cN classification					0.07		
cN0	112	35	147	38		1.0	
cN+	190	60	233	60		1.76	1.17–2.66
cNX	14	4	6	2		3.36	1.03–10.97
Period of diagnosis					0.01		
2004–2008	60	23	73	16		1.0	
2009–2014	326	77	243	84		0.48	0.35–0.76

*EAC* esophageal adenocarcinomas; *ESCC* esophageal squamous cell carcinoma; *dCRT* definitive chemoradiotherapy; *nCRT* neoadjuvant chemoradiotherapy; *OR* odds ratio; *CI* confidence interval

^a^The effects of type of comorbidity on treatment allocation were evaluated in separated models, which are adjusted for all variables in Table 2 expect number of comorbidities. Reference category for effects of type of comorbidity: No comorbidity

^b^Category unknown is not shown

### Association Between Comorbidity and Treatment

Patients with multiple comorbidities underwent more often dCRT (160/273 patients; 59%), whereas patients without comorbidities more often underwent nCRT plus surgery (142/211 patients; 67%; Table 2).

Multivariable logistic regression analysis confirmed the associations of age and comorbidities with type of treatment. Patients ≥ 75 years of age (odds ratio [OR] 8.58; 95% confidence interval [CI] 4.72–15.58) and patients with multiple comorbidities (OR 3.09; 95% CI 1.93–4.93) had a higher probability to receive dCRT than nCRT plus surgery. Regarding type of comorbidity and the likelihood to receive dCRT, the association was higher for the combination hypertension and diabetes (OR 3.80; 95% CI 1.97–7.32) and for cardiovascular with pulmonary comorbidity (OR 3.18; 95% CI 1.57–6.46; Table 2).

### Long-Term Overall Survival

**Fig. 2:**
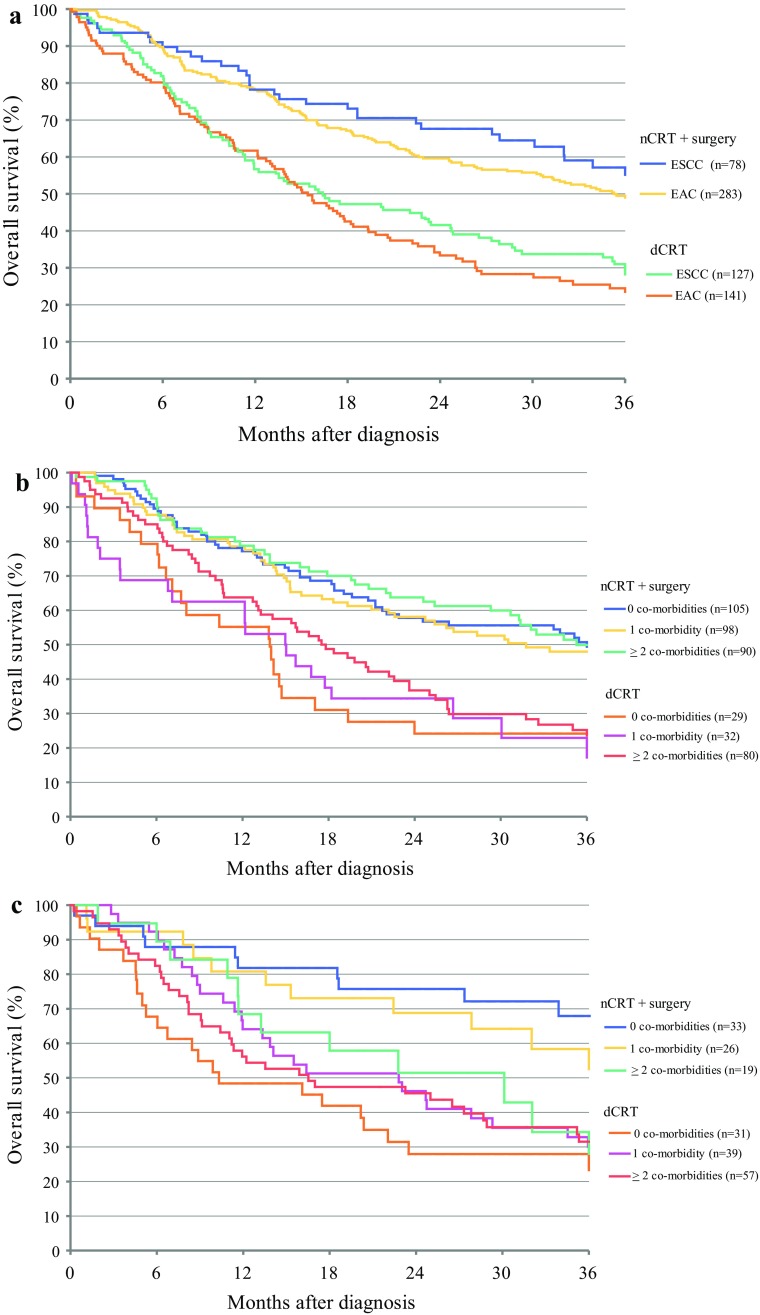
**a** Overall survival of patients (cT1N+/cT2-3,Tx, any N, M0) according to morphology following dCRT or nCRT followed by surgery (*n* = 205). Patients who died within the first 6 months after diagnosis were excluded from the analysis. **b** Overall survival of EAC patients (cT1N+/cT2-3,Tx, any N, M0) according to the number of comorbidities following dCRT or nCRT followed by surgery (*n* = 424). Patients who died within 6 months after diagnosis were excluded from the analysis. **c** Overall survival of ESCC patients (cT1N+/cT2-3,Tx, any N, M0) according to number of co-morbidities following dCRT or nCRT followed by surgery (*n* = 205). Patients who died within the first 6 months after diagnosis were excluded from the analysis

Two-year overall survival (OS) of all patients was significantly better following nCRT plus surgery compared with dCRT (61% vs. 38%; *p* < 0.01). Even after stratification for histological tumor type, the survival differences remained statistically significant (EAC: 60% vs. 33% respectively, *p* < 0.01; ESSC: 68% vs. 42% respectively, *p* < 0.01; Fig. 2a).

### Impact of Age and Comorbidity on Long-Term Overall Survival

Kaplan–Meier survival analysis showed that the 2-year OS was worse among patients with EAC who underwent dCRT compared with those who underwent nCRT plus surgery, regardless of the number of comorbidities (Fig. 2b). In contrast, the 2-year OS for ESCC patients with multiple comorbidities after dCRT (46%) was comparable to the 2-year OS (51%) following nCRT plus surgery (Fig. 2c).

Multivariable Cox regression analyses showed that EAC patients had a poorer prognosis following dCRT compared with nCRT plus surgery, irrespective of age and number of comorbidities (Table 3). Especially, among patients with cardiovascular diseases, hypertension or diabetes survival was poorer after dCRT.

**Table 3:** Multivariable Cox regression analyses to examine overall survival differences among patients who underwent dCRT versus patients who underwent nCRT followed by surgery according to age, number, and type of comorbidity, stratified for histology

	EAC			ESSC		
	*N*	HR of dCRT versus nCRT +surgery	95% CI	*N*	HR of dCRT versus nCRT + surgery	95% CI
Patients who died within 6 months after diagnosis were excluded to reduce immortal time bias						
Number of comorbidities*						
0 comorbidities	134	3.21	1.85–5.57	64	4.14	1.80–9.52
1 comorbidity	130	2.99	1.73–5.19	65	2.31	1.10–4.89
≥ 2 comorbidities	160	2.67	1.75–4.09	76	1.52	0.78–2.97
Age (year)**						
< 60	116	4.95	2.63–9.32	55	2.30	1.09–4.85
60–74	230	2.33	1.63–3.34	117	2.72	1.58–4.69
75+	78	2.17	1.09–4.30	33	0.73	0.13–4.14
Type of comorbidity^a^						
Cardiovascular diseases	131	2.32	1.42–3.77	67	1.68	0.83–3.40
Pulmonary	64	1.84	0.90–3.78	32	n.a.	
Hypertension	142	3.34	2.10–5.34	62	3.22	1.22–8.50
Previous malignancies	33	1.30	0.36–4.67	28	0.98	0.25–3.90
Diabetes	69	2.95	1.50–5.81	16	n.a.	

*n.a.* not assessed (too small number of patients), *HR* hazard ratio, *CI* confidence interval

*Adjusted for gender, age, tumor stage, and period of diagnosis

**Adjusted for gender, tumor stage, number of comorbidities and period of diagnosis

^a^Models for type of comorbidity were adjusted for gender, age, tumor stage, period of diagnosis, and number of comorbidities

In contrast, among ESCC patients with ≥ 2 comorbidities or age ≥ 75 years, OS after dCRT was comparable to OS after nCRT plus surgery. This was especially the case among ESCC patients with cardiovascular diseases or previous malignancies. However, ESCC patients with hypertension as the only comorbidity had a poorer OS after dCRT compared with nCRT plus surgery. The impact of pulmonary diseases or diabetes could not be assessed accurately due to the small number of patients (Table 3).

## Discussion

The results of this population-based study support the use of nCRT plus surgery in operable patients with EAC, which was associated with a better overall survival regardless of age, number and type of pretreatment comorbidities. The administration of dCRT was preferable in patients with ESCC with at least two comorbidities or age ≥ 75 years, because there were no differences in overall survival than with nCRT plus surgery in these patients. This was seen particularly among those with cardiovascular diseases or previous malignancies as their overall survival after dCRT was comparable to the overall survival for patients after nCRT plus surgery.

In the Netherlands, nCRT in combination with surgery is the standard potentially curative treatment for locally advanced esophageal cancer. This treatment potentially downstages the tumor and increases the radical resectability (R0) rate, which in turn reduces locoregional recurrences with improved long-term survival.^1^ Moreover, the regimen of the CROSS trial also showed control of distant disease beyond the first 24 months after nCRT, supporting a direct systemic effect.^25^

Of great importance for a prolonged survival is a pathological complete response following nCRT, which occurred in 49% of the patients with ESSC included in the CROSS trial and in 23% of those with EAC.^1^

In our study, 78% of the elderly patients were treated with dCRT and survival in elderly patients with ESCC was equal for both treatment modalities. Elderly patients are generally regarded as less suitable for surgery because of advanced age (≥ 75 years), comorbidity severity or decreased performance status. Moreover, dCRT seems a well-tolerated alternative for patients with EC who are not fit enough to undergo surgery.^1^,^7^,^11^,^12^,^17^,^22^,^23^,^26^,^27^ Nevertheless, selecting the appropriate treatment for elderly patients requires the presence a consulted geriatric physician in the multidisciplinary board.^28^

A relatively good outcome was reported after dCRT in selected groups of patients.^12^,^29^–^32^ Two studies have found a comparable OS after dCRT compared with surgery alone for patients with resectable ESCC.^11^,^12^ However, in these studies, survival differences were not investigated according to number and type of comorbidities. We found no significant difference in OS following dCRT or nCRT plus surgery in patients with ESCC having at least two comorbidities. This suggests that patients derive the same benefits from both treatment methods, although the type of comorbidity may have an impact on the outcome.

In patients with EAC, the standard approach of nCRT followed by surgery indeed resulted in a better survival, which also was found in the group with diabetes mellitus, hypertension, or cardiovascular disease. Tougeron et al. reported a more frequent use of dCRT in advanced staged EAC, in elderly patients and those with comorbidities of Charlson score ≥ 2.^13^ Despite selection bias may be present, survival after surgery was better compared with survival after dCRT (median overall survival 36.2 vs. 16.5 months; *P* = 0.02). Another study has found a significant improvement in median survival for patients with locally advanced EAC treated with nCRT followed by surgery compared with dCRT.^14^

These differences in treatment response between patients with EAC and ESCC may be associated with tumor aggressiveness and different carcinogenesis.^13^ Moreover, tumor site (distal vs. proximal) and pulmonary based differences with larger fields of radiotherapy in lower esophageal tumors also may play a role in outcome differences between EAC and ESCC following dCRT.^33^

With current radiation techniques, including intensity-modulated radiotherapy (IMRT), direct simulation based on 3D or 4D planning CT, respiratory gated radiotherapy, and intensity-modulated proton therapy the radiation dose can be accurately delivered with less damage to normal tissues.^15^,^33^–^36^ Moreover, in diminishing toxicity of chemotherapy regimens, the combination of carboplatin/paclitaxel has shown to be a good alternative or even the standard approach in dCRT, especially in patients with cardiovascular and pulmonary comorbidities.^37^

Our study has some limitations. First, the intent of curative treatment with chemoradiotherapy (primary dCRT or nCRT) was uncertain in this retrospective study. As patients with M1 disease were excluded, it was assumed that chemoradiotherapy was given with curative intent in all included patients. However, a small subset of patients were not fit enough or unable to undergo the planned surgery after nCRT and were treated with CRT alone or allocated to the dCRT group. This may lead to a less homogeneous group of patients treated with dCRT. Moreover, of the excluded EC patients who had surgery alone (Fig. 1), surgery could had been still a treatment options for some patients not suitable for chemoradiotherapy. As reported, a considerable number of these patients were not eligible for surgery due high age (> 75 years) and serious multiple comorbidities, the OS was worse after surgery alone in a previous analysis of potentially curable EC patients (*n* = 1,184) during 1995–2013.^2^,^3^,^19^ The 3-year OS in patients with EAC after surgery alone was worse but comparable among those with ≥ 2 comorbidities after dCRT (HR 1.07; 95% CI 0.72–1.60). The 3-year OS among ESCC patients after dCRT was comparable with those after surgery alone, despite the number of comorbidities, and even more favourable in those with pulmonary disease (HR 0.81; 95% CI 0.32–0.71).^38^ Second, limited information was given about the radiotherapy techniques and schedules of the given chemoradiotherapy. Since 2004, however, there was an increased preference for carboplatin/paclitaxel with less severe toxicity (6%) compared with cisplatin/5-FU (15%) as standard regimen in dCRT, especially in patients with cardiovascular comorbidity.^37^ Third, the impact of type of some comorbidities could not be assessed accurately due to a small number of patients. Moreover, information about the performance status was not registered for the study period. Furthermore, the accuracy of the diagnostic and staging methods used is unknown, while endoscopic ultrasonography was not always possible in patients with EC leading to unknown reported clinical T-stage in 15% of patients. Although out of the scope of this study, salvage surgery in solitary localized recurrences or persistent disease after CRT could be a curative option in selective cases. However, these procedures were not registered accurately at that time, because intensive follow-up was not commonly performed, and these procedures were then only performed occasionally in special centers.^19^

The strength of this population-based study is that the results are based on patients diagnosed in ten hospitals providing an overview of everyday clinical practice, rather than single-institution results in which patients are possibly more carefully selected.

In conclusion, neoadjuvant chemoradiotherapy plus surgery should basically be advised in operable patients with potentially curable esophageal adenocarcinoma regardless of age, number, and the type of comorbidities. Definitive CRT may be preferred in patients with esophageal squamous cell carcinoma having at least two comorbidities or being older than 75 years. For a better selection of patients, who may benefit from dCRT, prospective studies are needed.
